# Recovering the body in grief: Physical absence and embodied presence

**DOI:** 10.1177/1363459320931914

**Published:** 2020-06-07

**Authors:** Caroline Pearce, Carol Komaromy

**Affiliations:** The University of Cambridge, UK; The Open University, UK

**Keywords:** bereavement, body, grief, linear time, recovery, stage models

## Abstract

This paper addresses the complex issue of the embodiment of grief. It explores how a theoretical shift to the body has influenced scholarly literature about grief and bereavement. Despite this shift, we argue that bodily interpretations and experiences are undertheorised in western psychological literature on bereavement. Specifically, we argue that linear stage models of grief have encouraged the view that grief needs ‘working through’ in the mind, and not necessarily the body. We draw on empirical data from interviews with bereaved people undertaken in England to illustrate aspects of the embodied experience of grief that differ from how psychological grief theories conceive of the bereaved person’s body. Findings highlight the role of the bereaved person’s body in managing grief and how the absence and continuing presence of the deceased person is managed through embodied practices. We conclude that understanding grief as an embodied experience can enable the development of grief theories that better capture the complex negotiation between the psychological processes of grief and the materiality of bodies.

## Introduction

The bodily impact of grief has long been evident in research on grief and bereavement. Classic texts such as the longitudinal study of widows by Parkes (1970) highlighted the impact of bereavement on mortality and morbidity and research evidence demonstrates associations between bereavement and a range of physical conditions (Carey et al., 2014, Vitlic et al., 2014). Somatic symptoms such as sleep disturbance and changes in appetite are also documented in bereaved people at risk of developing complicated or prolonged forms of grief (Prigerson and Maciejewski, 2017). This research affirms the accounts of bereaved people who often emphasise both the physical and emotional pain of grief (Kelly, 2016).

Despite evidence of the physical impacts of bereavement, understanding the role of the body and embodiment in grief remains noticeably undertheorised (Brinkmann, 2017; Fuchs, 2018). Brinkmann (2017) argues that grief tends to be positioned as one of a number of potential risk factors where the body is the dependent variable. The focus is on how bereavement ‘gets inside’ the body and where grief manifests with the aim of understanding how to relieve and prevent physiological symptoms. For instance, neuroscientific research that aims to identify patterns of brain activity in bereaved people (O’Connor, 2005).

The limited theorisation of embodiment in grief may be one consequence of the dominance of psychological theories in grief research and practice: an explanation we explore in this paper. By viewing grief as an intra-psychic process, psychological research on grief has focused on the significance of the individual psyche, attending to the role of personality traits or coping and attachment styles on patterns of grieving. Bodily symptoms are acknowledged but commonly viewed as reflections of a disturbed mind. Clinical management of grief seeks to relieve physical symptoms primarily by psychodynamic or cognitive behavioural therapies that view grief as something to be ‘worked through’ mentally, and not necessarily physically. This explanation of grief recovery is rooted in Freud’s (1917) seminal ideas which established constructions of ‘normal’ and ‘abnormal’ grief. Subsequently, the development of stage models of grief consolidated the view that normal grief was a time-limited and individual psychological process, and popularisation of such perspectives meant the body became ‘lost’ in grief and bereavement literature (Tanner, 2006).

We argue, in line with Brinkmann (2017), that grief is not something that simply impacts upon the body but rather is performed or enacted by the embodied bereaved person. We agree with Tanner (2006) that an apparent disavowal of the embodied dynamics of grief reflects a cultural discomfort with the ‘rupture of autonomy’ that is manifest in both embodiment and the recognition of intercorporeality (Csordas, 2008). More broadly, this disavowal reaffirms the Cartesian divide between mind and body often repeated in psychological theories of the emotions (Hughes, 2000). Here, we contribute to explanations of grief that move beyond viewing the body merely as a site of somatic symptoms.

We draw on interview data with bereaved people in England to examine how grief is experienced at both emotional and physical levels and how the absence and continued presence of the deceased person is managed through embodied practices. We consider how participants’ accounts might indicate aspects of the embodied nature of grief that differ from how psychological grief theories conceive of the bereaved person’s body, challenging the notion of a time-bound linear recovery. The findings reveal how people experience the continued presence of the deceased person, highlighting how identities and agency can exist beyond the body.

We begin by discussing the significance of linear, stage models of grief for understandings of ‘normal’ mourning and recovery. The following sub-sections detail research that challenged these grief models, and which foregrounded the relational and embodied aspects of bereavement.

### Grief as a time-bound, linear process

Early academic studies of grief (Gorer, 1965; Lindemann, 1944) built on Freud’s (1917) ideas on mourning and melancholia to proclaim successful mourning as a psychological process achieved by ‘letting go’ and ‘detaching’ from the deceased person. This understanding of grief subsequently became embedded in bereavement research and practice. The dominant approach to grief study held by psychologists and psychiatrists based on phases, stages and tasks has justified therapeutic interventions (Bowlby, 1980; Kübler-Ross, 1970; Parkes, 1970; Worden, 1982). Such studies described grief as a process and their authors did not necessarily intend for stages to become prescriptive. Regardless of this caution however, the linear-staged model of grief became the defining theory in bereavement care and, despite substantial criticism of the lack of supporting empirical evidence (Corr, 2018) it continues to have considerable lay and clinical traction (Kenny et al., 2017).

Certainly, identifying people in need of help is well intentioned and some bereaved people report finding the stage model helpful. However, while stage models of grief serve to normalise grief as a process, they also locate experiences that fall outside the normal range as potentially pathological or deviant. The notion of bereavement recovery is contested (Rosenblatt, 2008), and linear-staged grief models reflect broader societal expectations around recovery. For instance, that grief is time-limited and emotions should be managed appropriately (Kenny et al., 2017). How long grief symptoms persist following bereavement is decisive in determining whether a bereaved person’s grief is judged as normal or presents a risk to mental health (Boelen and Prigerson, 2012). The time frame for ‘normal’ grieving may not always be specified but, as Kenny et al. (2017) describe in their study, seeing grief as time-limited remains a dominant perception among bereaved people.

The continuing significance of temporal frames to measure normal and abnormal grief supports the idea that bereavement, at least in western societies, poses a disruption, or as Small (2001) described it, a ‘fissure in modernity’. It also implicates a ‘docile’ grieving body with defined boundaries (Small, 2001). Wambach’s (1985) study revealed how widows used the stage model to monitor their progress against a timetable. Though women’s experience would often contradict the stages, the process was so naturalised most widows did not realise the grief process had an origin and was a social creation. In this way, the stage model had become what Foucault described as a ‘technology of the self’ (1975); a means of self-regulating bodies.

The scientific approach to mortality, which is reflected in the stage model of grief, has sought to resolve the disruption bereavement presents by affirming the belief that life and death are clearly separate (Howarth, 2000). This division between life and death, is not a fact, as Howarth (2000) argues, but rather a false boundary borne out by a modern need to segregate and classify. Bereaved people appear to embody this divide, posing a problem in a society orientated around life and troubled by death. Research that has acknowledged how bereaved people continue relationships with the deceased has posed a challenge to the perception of a life/death divide.

### Continuing bonds after death

The end of the twentieth century witnessed a shift away from psychological and psychoanalytic theories toward social models of grief. Postmodernist theories emphasised the role of individual, reflexive meaning making and narratives, and how people continue bonds with the deceased person (Neimeyer, 2005; Klass et al., 1996). These theories highlighted the social and interdependent nature of human relationships, opposed to the individualistic psychological model. Klass et al. (1996) rejected Freud’s (1917) theory stating that it was based on a faulty view of the social self in which people were seen as separate from one other. Instead, they proposed ‘that it is normative for mourners to maintain a presence and connection with the deceased’ (1996: 17). Challenging the linear notions of time implied in stage models, the ‘dual process’ model of grief acknowledged that bereaved people move back and forth in their grief, alternating between periods of focusing on the deceased person and periods of avoidance and distraction (Stroebe and Schut, 1999).

Such theories did not necessarily challenge sequential time as part of modernity, nor impact on the belief in death as biological fact (Howarth, 2000). Árnason (2012) and Leichtentritt et al. (2016) argue that Klass et al.’s theory continued to overlook the embodied and relational aspects of continuing bonds reaffirming the assumption that life and death are clearly separate. Recent research on continuing bonds has emphasised how post-mortem relationships are managed through situated and embodied social practices such as memorialising the dead as part of an embodied process of meaning-making (Valentine, 2013). Social practices are also the focus of Maddrell’s (2013) work on informal public memorials. Developing the concept ‘absence-presence’ Maddrell argues that absence becomes a dynamic ‘absence-presence’ located in material spaces, not just in the psyche. This research highlights how continuing bonds are socially performed through embodied practices that serve to mediate between private and public, and between the living and the dead.

In recent decades psychological bereavement research has focussed on developing psychiatric categories of grief (Boelen and Prigerson, 2012). Despite the shift toward social theories of grief, research on ‘complicated’ and ‘prolonged’ forms of grief has foregrounded again the idea that grief is a time-bound and linear process, reinforcing precisely the type of modernist measurement culture that post-modern scholars actively resisted. Here, we argue that contemporary grief theory that is based on death as a biological fact, located in linear time, fails to adequately capture the continued agency of the deceased person.

### Beyond the body

While it might seem to be an empirical truth that the dead body is no longer able to communicate its selfhood in any embodied way, its identity continues through its social presence and researchers have examined different ways the body and identity of the deceased person endures (Hockey and Draper, 2005; Hockey et al., 2010). As Howarth (2000) argues, constructing bodies as bounded and separate from other bodies fails to acknowledge a continued social presence that is separate from material bodies. Hallam et al. (1999) challenged the idea that agency is body-based arguing that after the disposal of the flesh, the dead have a continued hybrid existence. This poses the question of how the self survives when the body is gone, drawing attention to how the social existence of the deceased person is not only psychological but can be experienced in the body of the survivor, through the incorporation of physiological sensations.

Studies into ‘sense of presence’ experiences highlight how some bereaved people report a range of sensory experiences including seeing, hearing or experiencing smell or touch and or vivid perception of the deceased person’s presence (Keen et al., 2012; Steffen and Coyle, 2012). Bereaved people generally find these comforting and helpful, but they can be experienced as negative and unwelcome (Steffen and Coyle, 2012). From a professional perspective, debate continues on whether sensory perceptions are ‘healthy’, or a potential risk factor for complicated grief (Field and Filanosky, 2009). Bennett and Bennett’s (2000) study of experiences of the presence of the dead revealed how bereaved participants would use both materialist and supernaturalist discourses to make sense of their experiences. Findings revealed how participants’ narration of their experience drew on modern rational discourse that classified dead bodies as separate from living ones, and participants’ own fears of being considered delusional.

Non-western grief rituals and practices provide a contrast to western norms about the role of the body in death and disposal. For instance, in some non-western cultures keeping the dead body of a family member in the home for several months or longer is a traditional custom, such as the Torajans in Indonesia who also periodically bring out deceased people from their graves to give them new clothes and gifts (Bennett, 2016). By contrast, stories of bereaved people who keep the body of a deceased person at home feature in western media shrouded in a sense of disgust and repulsion (for example see Collins, 2012). While such practices are diverse, they highlight how western bereavement literature fails to capture how the body of the deceased person and the bereaved person is integral to many death and mourning traditions (Martin et al., 2013).

Ribbens McCarthy and Prokhovnik (2014) expand on ideas discussed so far to explore how it is possible to speak of caring after death within a western secular framework that views death as the absolute loss of a person. They argue that within a framework that assumes a mind/body and life/death divide, it is unclear what happens to personhood following bodily death. Countering this view, they suggest that while the biological body dies, the embodied relationship does not. The felt material presence of the dead person remains as well as the ‘us’ formed in the relationship between the two bodies. The persistence of an enfleshed and material connection continues in the body of the living person as an ongoing embodied presence. Significantly, they challenge the notion of ‘internalising’ the dead person that is implied in many grief theories, including continuing bonds, contending that it remains predicated on the mind/body split by psychologising and locating the deceased other within an individual’s psyche. Instead they recommend the need to move beyond the binary thinking that is implied in grief models to consider how people maintain a material and embodied relationality after death.

We argue that the experiences of the participants in this study challenge the dominant logics about bereavement as a time-limited and disembodied process. Rather, the body and embodiment play an important role in the experience of bereavement through physiological sensations, managing grief through routine physical activities, and sensing the presence of the deceased person.

## Methods

The data in this paper were collected as part of a PhD study conducted by the first author, and supervised by the second author (Pearce, 2019). The study aimed to interrogate the notion of recovery from grief following bereavement, with a particular focus on understanding the experience of ‘not recovering’ or becoming ‘stuck’ in grief, and how such an experience is socially shaped and constructed. Fieldwork was carried out in 2014-15. Ethical approval was gained from The Open University Human Ethics Committee.

Bereavement organisations, including national charity Cruse Bereavement Care and St Christopher’s Hospice, were contacted to assist in recruitment. We used a snowball sampling approach. The participating organisations invited relevant individuals to participate via information sheets, mailing lists and staff newsletters. Information about the research was also posted on social media groups and websites. The criteria for participants consisted of being aged 18 or over and having experienced a form of bereavement. No restrictions were placed on the type of or time since bereavement in order to capture a range of experiences.

Nine participants (see Table 1) had experienced different types of bereavement and the time since bereavement at interview varied from 9 months to 26 years. The participants included three men and six women, with six bereaved by the death of a partner. The sample is not intended to be representative and we acknowledge the limitations of our findings, drawn from a small sample. However, we believe that the interview data provide rich narrative accounts of bereavement and contribute to understanding the undertheorised role of the body and embodiment in grief.

**Table 1.** List of participants.

Name	Age	Type of relationship	Time since bereavement
Anne	46	Spouse/partner	4 years
Tania	41	Spouse/partner	2 years
Laura	51	Spouse/partner	2.5 years
Saadhia	30	Parent	4 years
Jamie	34	Sibling and Parent	10 and 18 years
Paul	64	Spouse/partner	1 year
John	63	Spouse/partner	9 months
Rose	63	Spouse/partner	26 years
Helen	64	Parent	8 years

The interviews were conducted by the first author (CP). Before the interview, each participant was provided an information sheet about the research and asked to sign a consent form. Participant’s names and any identifying places have been changed to protect anonymity. Interviews were recorded on a digital recorder and conducted at the participant’s home or a convenient public venue. Interviews were semi-structured and aimed to capture participants’ experiences of bereavement and their thoughts on the notion of recovery. To begin, each participant was invited to share details of their bereavement. Most needed little prompting and narrated in detail their experiences of bereavement and grief. The interviews became less structured as CP realised the interview provided a space for the participants to narrate their experiences of grief. Interviews lasted from one to two and a half hours.

The interview setting produces a ‘situated’ form of knowledge and in qualitative grief and bereavement research the ‘situatedness’ of knowledge is especially pertinent when the fact of death and mortality is something ‘we’ are all ‘inside’ and part of (Woodthorpe, 2007). Rowling (1999) suggests grief and bereavement researchers should strive to be neither too ‘in’ nor ‘out’ but ‘alongside’ participants. Acknowledging the situatedness of the knowledge involved the first author being reflexive to her own biases. Being ‘reflexive’ does not promise objectivity, it is rather a strategy that helps to reduce harm to the participants and being clear about one’s position as researcher. This reflexivity was something that required constant renegotiation in the fieldwork process and was integral to achieving transparency in the influences on data.

The clarity of these boundaries was at times challenged by the emotional nature of the interviews. As Parkes (1995) notes, bereaved people and especially newly-bereaved people are vulnerable, often experiencing strong emotions. Following Renzetti and Lee (1993) we also take the view that all research has the potential to be sensitive, and so sensitivity should not be used to describe only particular groups or approaches to research. One example from our fieldwork demonstrates the responsibility required on behalf of the researcher when conducting potentially sensitive research. Following an interview, a participant contacted the first author clearly distressed and wanting someone to talk to. CP responded by being empathetic but reaffirming the boundaries, making it clear that she could not be accessible to him in this way. She sought advice from her supervisors and informed the gatekeeper, who had put her in contact with the participant, of the incident.

Maintaining clear boundaries was therefore not always straightforward. At times, it seemed to the first author that what was ‘theirs’ and what was ‘mine’ could be difficult to disentangle. For example, conducting the interviews would lead CP to reflect on the inevitable loss of those close to her. CP thus saw her own body as another participant immersed in the space (Ellingson, 2012). Monitoring her own emotional responses to the interviews was one means of guarding against the potential exploitative effects of the interview process.

The interviews were transcribed by the first author and then analysed thematically by coding the content of the data (Charmaz, 2004). The coding was completed by the first author who read the transcripts several times to note repetitions and recurrences to construct an initial long list of codes. Discussions with the second author assisted in the development of the overarching themes. The long list was refined to three overarching themes that were then separated into sub-themes. Two of these themes - practices and identities – included significant emphasis on embodiment which is discussed in this paper.

Analysing the stories people tell about themselves and their losses, we noted how participants would often speak about grief in metaphors. For example, grief was often described as a ‘dark’ place and a sense of ‘going downhill’. These metaphors provided an insight into the sensory experience of grief, used as a means of grasping at this thing ‘grief’ which kept eluding concrete terms. Metaphors structure and organise experience and tend to have coherence within a larger cultural system. The choice of metaphor may serve to highlight some aspects of experience and hide others (Lakoff and Johnson, 1980). It occurred to us that this also had implications for the extent to which the embodied experience could be captured in the format of an interview. Transcribing the interviews and typing speech into words on a blank page introduced a level of distance in the participants accounts. Participants communicated not only verbally but also through mumbles, gestures, or silences. This level of communication was difficult to translate into the transcribed text.

Therefore, this has implications for the presentation of our findings. We contend that grief is made sense of through the body, but the interview setting requires people to use language to express their ideas. Language and culture filters bodily experiences, it is not possible to report ‘pure’ bodily sensation (Ellingson, 2012). This is further constrained in the interview setting which demands a rational and articulate interviewee. We acknowledge this tension as we seek to convey how bodies were made present in the participants accounts below.

## Findings

The participants accounts presented in this section extend from detailing the impact shortly after the death to the longer-term negotiations of relationships and identities, suggesting the enduring and changing nature of the impact on and role of the body in bereavement.

First, we begin by focusing on the management of the deceased body. Second, we present how bereavement affected how participants experienced and inhabited their own body. The further two themes describe how the bereaved participants managed both the absence and presence of the deceased person.

### Confronting the deceased body

Witnessing the dead body was for all participants a distressing and at times haunting experience. Anne described her husband’s dead body as ‘an envelope’ with the ‘contents gone’. Confronting the body of the deceased person was resisted by some of the participants and Paul explained why he did not want to see his wife’s body: ‘I didn’t want to see her as she was’. But while fearful, he found viewing helpful as the preparation of her body had removed the visible signs of her illness: ‘it obliterated how she was in hospital’. The impact of the person’s declining and dying body could be powerful. Jamie described how witnessing the deterioration of his mother to cancer was a memory he later sought to ‘block out’ by heavy drinking.

Saadhia also described a fear of seeing her mother’s body. While she felt uneasy being with a dead body, the fact it was her mother seemed to dissipate her fear:

We got her into the car, and then we took her, we took her to the mosque. I was sitting in the back with her and I was thinking I’ve never done this (. . .) if it was someone else I would have been scared because death is a thing I used to be scared of and seeing dead people it was like oh my god in films you just (gasps) and I was there with my Mum and it just didn’t feel right

Saadhia detailed further her role in the management of her mother’s body:

I had to close her eyes and cover her head, take all her jewellery off and straighten her hands and legs. It was stuff I’d read, Islamically what we’re supposed to do when someone passes away.

(. . .) she’s there ready to have her wash and I couldn’t wash her because you’re not allowed if a woman’s menstruating, you’re not allowed to touch the deceased. So I gave all my kisses and hugs and everything before everyone else took over, and I just watched from a distance.

Apart from Saadhia’s description above, the participants accounts did not include detail of the management of the deceased body. This is likely because most of the participants relied upon professionals to prepare and dispose of the body. In Saadhia’s case she was guided by religious practices which provided specific tasks concerning the dead body that she had to complete. However, the full completion of these embodied practices was prevented by her own menstruating body. This meant that she, similarly to the other participants, oversaw the management of the dead body at a distance.

### Inhabiting the bereaved body

In the immediate aftermath of bereavement participants described feelings of shock and numbness. As this numbness subsided, the individual bodily effects of grief became evident, described as physical pain. Participants used descriptors such as ‘spiky’ and ‘stabbing’ to express these sensations. John described a ‘physical feeling of sickness in my stomach, a griping sort of feeling’. Anne, whose husband died suddenly, detailed in visual terms the experience of grief:

I’ve explained about the physical pain and I always thought that was a metaphor; it’s not, it’s physical. My whole body cavity just screamed in pain, and even now when I’m stressed my sternum feels like all my tendons are pulling off it physically. I just had this view of the inside of my body being this black and splattered cavity where my heart and soul had just splattered into a million soggy pieces.

Anne remarked how she had thought the pain of grief was metaphorical, suggesting the pain was unexpected and underlining the assumption of grief as a purely emotional experience. The use of verbs ‘screamed’, ‘pulling off’, ‘splattered’, conjure an arresting and violent portrayal of the physical impact of grief. Anne acknowledged that she liked to use metaphors and used them often to describe her feelings. There were evident limits to how much the participants could convey the sensory experience of grief through the format of an interview. Participants often emphasised how they found the experience hard to quantify and articulate in words.

Participants described experiencing a number of bodily symptoms such as sleeplessness, difficulty eating, and changes in weight. For example, Laura experienced weight gain, which she linked to a lack of confidence following her husband’s death:

I’m still not confident about going out. Because [Laura’s husband] was always there so I would go places with him no problem and he was a very quiet man but he was big! Six foot, built like the side of a barn, stood at my shoulder, looked ferocious (. . .) It gave me the confidence to be myself. And I’ve lost my confidence in being myself. (. . .) Because I have no grounding. I think becoming a widow highlights your inadequacies. (. . .) Because you’re so raw.

Laura’s sense of confidence was tied to being in a couple which in turn was intimately linked to the bodily presence of her husband. His absence affected her own sense of body size. The loss of feeling ‘grounded’ suggests the support and reassurance that can be gained from a relationship. It also indicates how in bereavement people may experience a change not only in the perception of their own embodied self (for example feeling like your ‘inadequacies’ are exposed) but also in how they perceive the relation between their body and the environment. In other words, bereavement shifted the participants’ perspective on how they experienced and inhabited their own body.

This shift of bodily orientation in space (Krasner, 2004) was evident in how the participants described experiencing the space of the home, previously inhabited with a partner or family member, and was amplified for those who had been long-term carers of their partners or parent before they had died. For Paul, the death of his wife was particularly marked as he had spent the majority of his adult life married and living with one partner. The person’s absence demonstrated how much space as well as time had been occupied by the work of caring, as Paul described:

She became virtually supine for about two years (. . .) the routine you had, getting up and doing things and looking after them and making sure this and doing some washing every day and washing her and cleaning up and making sure she’s okay and using the commode, clearing that up multiple times a day (sighs). And then when that’s not there

Paul described how his daily routine was orientated around the care of his wife’s dying body. While his days had once been filled with tiring physical work, he was left with an absence all the more acute in that he had lost his wife and a daily routine that had provided a meaningful sense of time and purpose. The absence of the body by which one was once orientated and constituted left participants uncertain about how to relate to their own environment, bodies, and identities. Physical activities were used to manage these feelings, as we describe next.

### Managing absence

All participants, including those who were not carers, expressed a feeling that the centre of their life was lost and with it the ‘incentive’ or ‘motivation’ to carry on living. This was demonstrated in what they experienced as an inability to complete tasks and activities:

I start stuff and don’t finish it, so the house is full of quilts and knitting (. . .) I don’t finish anything. It’s too much effort to finish it, I get bored, and I have no motivation to move forward (. . .) There’s nothing, there’s no reason to my life, I go to work I come home I eat I do the things I’m supposed to do. (Laura)

As time went on, participants described how household and intimate work took on importance, particularly for Paul and John who were retired. Paul described how following the death of his wife he felt he had nothing to do, and so walking his dogs helped create a routine:

I make myself get up in the morning have a little bit of a routine, basically because of the dogs to be honest (. . .) so I have to get up and I get up at a certain time and do things but apart from that, apart from the fact I go out and do some shopping every day or make myself go out, that’s really, that’s it, I’ve got nothing. Nothing to do.

Whereas Anne described the period following her husband’s death as one where she felt almost compelled to take on projects, experiencing what she described as an ‘enormous amount of energy’:

I call it terrible energy, because it is terrible, you cannot sit still, you’ve got to drive and do things all the time, because as soon as you stop, it just, it’s like out running a tidal wave. And I have run and run and run.

Many participants tried to keep ‘busy’ by engaging in practical activities to manage their feelings of grief. While these activities did not necessitate the kinds of focused reflection and emotional ‘work’ involved in bereavement counselling, participants found that these embodied practices positively impacted their emotional and mental wellbeing. As Paul described it, physical tasks such as dog walking and shopping provided him with a ‘need’ to get up or ‘make’ himself go out and so structured his time. Similarly, John, following his wife’s death, talked about the need to find motivation in the form of a hobby:

I’ve been trying to help out with my son and my granddaughter (. . .) just the sheer logistics of nappies, feeding, burping and all the rest of it is a full-time occupation. And then along with trying to look after myself and keep my place, my house tidy and up to scratch, that in itself is busy, all told, that’s quite, quite busy.

Though he had never ‘had much of a hobby’, finding some activity suddenly had taken on a crucial importance in John’s life now that his wife was no longer with him. For both John and Paul it was sporting activities, active hobbies or work that became important in order to manage the experience of absence and grief. These activities helped provide a routine, a temporal structure to each day. The management of grief, for John and Paul, was not just ‘emotional’ work, rather they believed that physical activity could provide a mode of recovery. Possibly there is a gendered aspect to the finding that male participants Paul and John felt inclined to engage in social or practical activities as a form of coping. It also reflects how both were in retirement and did not have the external structure of work to manage their time. The other male participant in the study, Jamie, who was much younger than Paul and John and in his early 30s described quite a different experience.

Jamie’s two experiences of bereavement, of his sister and his mother, had a profound impact on his life. He had experienced periods of unemployment and homelessness that meant his life was much more precarious than the other participants. Jamie explained that he had difficulties with alcohol which had developed following the death of his mother. The way in which Jamie described the impulse to drink as a means to ‘block out’ and ‘forget’ shared some similarity with how John, Paul and the other participants would emphasise the importance of ‘diversions’ from their grief. For example, Paul and John described hobbies or household chores as ‘beneficial’ distractions for them both, as it enabled them to stop thinking about themselves or their wives, providing in effect a ‘diversion’, from their grief, as John explained further:

I suppose in effect that’s been beneficial for me because it’s, there’s always been something to think of apart from me thinking of myself and my wife you know (. . .) and I think that’s the main thing for me is if I can keep busy then I can take my mind off my problem.

These excerpts demonstrate how the experience of grief gave rise to a need to busy oneself; a strategy to counteract the absence of the deceased person. Participants described a struggle to figure out what to do with themselves, of doing things one is ‘supposed to do’ but with no motivation or desire: what appeared as a sense of suspended agency. Activities were used instrumentally as diversions and the physical effort of going out, of bodily activity, appeared to enable the mind to not focus on thoughts and memories of the deceased person, and even to act as a form of recovery. Finding new hobbies or simply getting out of the house may also have increased opportunities for social interaction, reducing possible feelings of isolation.

### Managing presence

Participants described experiencing the continued presence of the deceased person. For example, both Paul and Saadhia described hearing the voice of their deceased partner and mother. As Saadhia described:

I hear her calling me, she used to always say Saadhia, Saadhia! (. . .) the whole night, I was hearing her calling me. I just didn’t want to believe she was gone, I just wanted to believe she was there (. . .) Eventually I went to my doctor, and then I told my doctor this is what I’m feeling and I keep hearing my Mum calling me and I think it’s real.

Participants also described taking on personality traits of their deceased partner. Tania explained how she had adopted some qualities of her husband:

[my husband] was always the very calm one in our relationship, I was always very feisty and very panicky . . . So, I think I’ve taken on, I’m a lot calmer . . . I’m a lot more relaxed and I think that his qualities were like that.

After her husband’s death Rose acknowledged that she began to embody his qualities and described how other people had told her she sounded, ‘just like what Simon [her husband] would have said’. Rose explained that she also regularly spoke with her husband and would script his part of the conversation and play both roles:

If I want to talk to anyone I can sit there, and I know his reactions to conversations so much, so I know exactly how he would answer things. So sometimes I would come home upset and I would go ‘Simon what do I do now?’ and I feel him just say right okay, we’d sit down let’s go through what is the problem and I would hear his thoughts.

The partnership Rose had with her husband continued for 26 years after his death, and the presence of her husband appeared to be a helpful companion. Rose described that Simon was ‘brilliant at finding things’ and would provide directions when driving. Rose remarked that she felt a closer bond with her husband in his death than when he was alive and in her own words Rose credited this ongoing relationship as the reason she had not ‘really grieved’. In this respect, the continuing intercorporeality of Rose’s relationship with her husband illustrates Ribbens McCarthy and Prokhovnik’s (2014) argument that the personhood of the deceased person continues to exist through the body of the remaining partner. However, the way Rose narrated her relationship was more complex. While she said it felt as if ‘we’re still together’ she also described the peace she felt being her ‘own person’. Further, Rose acknowledged how her role had changed since her husband’s death. Rose was clearly comforted by the on-going embodied presence of her husband, yet she also experienced more freedom in his absence.

Rose’s example suggests that after death, the relational identity one had inhabited in a partnership might continue, but that partnership also continues to change and develop. As Rose described it, she could speak to her husband as and when she needed. Maintaining control meant, rather than becoming overwhelmed by memories or hearing voices, the presence of the deceased person and the relationship could potentially be shaped to one’s choosing. The participants’ interaction with household objects and possessions illustrated how they negotiated this continuing presence. For Paul, everyday objects took on new resonance in his wife’s absence and he struggled to keep the house tidy or clear his wife’s possessions:

And people say, “oh why don’t you get rid of them why don’t you do this and why don’t you do that”, I can’t (. . .) She’s got loads of clothes, loads of things, but everything is, the way I look at it, it’s like a betrayal. To me, you know.

John, too, after the death of his wife faced a similar struggle living in the house unchanged from when his wife was alive. He acknowledged that in keeping his wife’s possessions he was ‘still hanging on to her’:

I’m just, I’m not, I don’t want to lose her. And I know I have to (. . .) it’s sort of like I don’t want to lose her yet. And you think, at other times I think to myself, “Oh, that’s being silly” but I just, well it doesn’t hurt anybody just leave it as it is, it’s only going to affect me so why worry about what other people are going to think.

The interaction with material objects makes tangible the ongoing negotiation of managing the deceased person’s presence. Holding on to objects could be a way of holding on to the person who had died, yet conversely, the rejection of the home could be a rejection of the influence – positive or negative – the deceased person may have had over the home. Following the death of her husband, Rose began to hoard objects. Yet unlike Paul and John, Rose portrayed a sense of indifference rather than attachment to the accumulation of objects in her home. Rose contended her hoard was a result of the dominant role her husband had played in their home. By hoarding in her husband’s absence Rose was reclaiming the space as her own, leaving only enough space for her husband that she could manage comfortably.

## Discussion

We have argued that the psychodynamic theories that underpin how grief is interpreted tend to position grief as something external to the body, rather than intrinsically embodied. The bereaved person has to work through grief to resolution, whether freed from grief or internalised as part of a new identity. Rather than grief being something that merely impacts on the body, we have sought to demonstrate how it is enacted or performed.

Viewing the body in bereavement as a ‘risk factor’, or the dependent variable, ignores the relational dimensions of grief, that we contend are felt, expressed and performed through the body of the bereaved person. The bodily pain of grief described by the participants demonstrates the significance of the materiality of the body in how grief is experienced. Although many of the participants struggled to articulate this corporeal impact, we found that the body of the bereaved person was central to managing both the absence and presence of the deceased person. For most participants the visceral impact of bereavement tended to subside yet a shift in how they inhabited and experienced their own embodied sense of self remained.

The participants accounts included little detail of the management of the deceased body. This may evidence the professionalisation of death in western societies but also reflects the interview questions which tended to focus on the experience of grief. However, we argue that the deceased body and the embodied practices of the bereaved person are still evident in different forms. The ways in which the participants relied upon practical and social activities could be considered as a form of embodied ritual that helped to relieve the disruption of bereavement and make time feel ordered and thus meaningful once again. Similar to the findings of Valentine’s study (2013), the practices of continuing attachments to the deceased were not something that simply happened as a result of a psychological state but arose out of social action. The emphasis on establishing a routine and the desire to ‘do’ something, also reflects how bereavement, like other life events, is increasingly managed through as a project of the self with tasks to ‘work through’. Feeling compelled to find a hobby to structure one’s time could be interpreted through this temporal frame that obligates individuals to govern their time appropriately and efficiently (Kenny et al., 2017).

It was apparent that the absence of the bereaved person could encourage physical activity but also reduce it, or make it feel impossible. In this way physical capacity was affected by loss but also physical activities were believed to provide a means of resolution through a form of agency. John stated that he believed taking up a hobby would help resolve his grief over his wife’s death. And yet while the body was present in these activities, physical actions were utilised as a way to distract oneself from feelings, thoughts and emotions, and other physical expressions like crying. Some of the physical movements described thus appeared curiously disembodied in that they were performed as a resistance to, or devoid of, feelings and emotion.

The body could both be a means of ‘recovery’, and also a site of resistance to, or distraction from, a linear recovery process. The dual process model of grief (Stroebe and Schut, 1999) captures a similar mode of alternating between focusing or avoiding one’s loss. Here we emphasise the embodied nature of such action. Further, we suggest this action may not always be as intentional as the dual process model implies. Rather the participants described a situation of suspended agency. Engaging in activities was functional, a means to manage time and space, and not necessarily enjoyable.

Issues of identity came to the fore in how participants managed the absence and presence of the deceased person. This was especially marked for those who had lost a long-term spouse and/or had been the caregiver of the deceased person. The identity of the deceased person continued to change after death as it inhabited the living body of the bereaved person supporting Hallam et al.’s (1999) argument that identities come to be inhabited, within and beyond the body. The ways in which participants interacted with the home space and objects made tangible this process. Gibson (2004) utilised the psychoanalytic concept of ‘cathexis’ (Freud, 1917) to portray how bereaved people held on to material objects, not only psychic objects, as a mode of transitioning through their grief. Objects of the dead, Gibson argued, carry powerful symbols of their presence. In the data presented here, holding onto objects served as a means to ‘hold on’ to the deceased person, as John acknowledged. But objects could also be used as a means of resistance to the deceased person’s presence, as in the case of Rose.

Overall, however, attachment to objects appeared ambivalent, reflecting a sense of ambivalence in the continuing relationship with the deceased person. This was most evident in Rose’s account where there appeared to be a difference in the embodied and physical presence of her husband. In his death, Rose found comfort from embodying his qualities yet also experienced more freedom in his absence. This suggests a distinction between embodiment of a person and managing their presence. This finding also resonates with Maddrell’s (2013) research on memorialisation and the concept of ‘absence-presence’ which emphasises how the continuity of the deceased presence arises from the relational tension between the physical absence and emotional presence (a sense of still being there).

## Conclusion

Grief models and theories perpetuate a silent but assumed body, one that is contained and bounded despite challenges from scholars in feminist traditions and death studies. Our aim has been to bring into view the embodied experience of grief and bereavement. Though often hidden in psychological bereavement theory, the body of the bereaved person is left open to external interventions that seek to guide the bereaved person through a time bound linear recovery process. To destabilise this assumed normative body, the field of grief and bereavement research needs to further engage with body studies to better appreciate not only the physical impact or bodily symptoms but how identities and agency might exist beyond the body. This requires acknowledging the multiple discursive bodies of the bereaved and deceased person.

Recovery from grief is viewed as psychological work, here we suggest that everyday embodied activities can provide meaning and bring about a sense of recovery. We also argue that bodily practices can be used as a mode of resistance to normative societal expectations around grief, challenging time-bound tasks and stages. Psychological understandings of grief tend to reduce the body to something that can be impacted upon, leaving limited space to understand how the body is expressed and experienced in grief. While bereavement is a universal event, acknowledging the role of the body and embodied presence can enable understanding that people come to inhabit the experience in multiple embodied ways.
